# Prevalence, Species Distribution, and Related Factors of Fish-Borne Trematode Infection in Ninh Binh Province, Vietnam

**DOI:** 10.1155/2019/8581379

**Published:** 2019-07-30

**Authors:** Anh Kieu Thi Tran, Hoa Thuy Doan, Anh Ngoc Do, Van Thi Nguyen, Su Xuan Hoang, Huong Thu Thi Le, Hoa Thi Hoang, Nam Hoang Le, Quyen Bao Thi Le, Tran-Anh Le

**Affiliations:** ^1^Paediatric Department, Vinh Medical University, 161 Nguyen Phong Sac, Vinh, Nghe An, Vietnam; ^2^Ha Noi Police Hospital, Ha Noi, Vietnam; ^3^Department of Parasitology, Vietnam Military Medical University, 160 Phung Hung, Ha Dong, Ha Noi, Vietnam; ^4^Department of Microbiology and Pathogens, Institute of Biomedicine and Pharmacy, Vietnam Military Medical University, Ha Noi, Vietnam; ^5^Department of Microbiology and Biology, Ha Noi University of Pharmacy, 13 Le Thanh Tong, Ha Noi, Vietnam; ^6^Fundamental Medicine Department, Nam Dinh University of Nursing, 257 Han Thuyen Street, Nam Dinh City, Vietnam; ^7^Ninh Binh Centre for Disease Control, Le Thai To Road, Nam Thanh Ward, Ninh Binh City, Ninh Binh province, Vietnam; ^8^University of Science, National University of Ha Noi, Vietnam

## Abstract

*Background. Clonorchis sinensis/Opisthorchis viverrini *and minute intestinal flukes (MIF) such as* Haplorchis pumilio and H. taichui* are fish-borne trematodes (FBT) that may coexist in regions where local people have a habit of eating raw fish like Vietnam. Responses to FBT should be verified according to the data on the distribution of these flukes. This study aims to explore the prevalence of different species of FBT and related factors among local people in a northern province of Vietnam.* Methods.* A cross-sectional study was conducted in Kim Son and Yen Khanh districts, Ninh Binh province, between March 2016 and March 2017. Four hundred people aged 15 years or older were interviewed and gave stool samples. The FBT eggs in faecal samples were enumerated by modified formalin-ether technique and identified by sequencing of the second internal transcribed spacer (ITS2) region.* Result.* Among the 400 persons, 19.5% were infected with FBT. On univariate analysis, eating raw fish was the main risk factor (odds ratios (OR)) of 6.769 (95% confidence interval (CI) of 2.655–17.259) followed by being of male gender (3.994 (CI95% 2.117–7.536)) and drinking alcohol (2.680 (CI95% 1.440–4.986)), respectively. There was no risk of increased infection among those living at home without hygienic latrines, those living close to rivers or having ponds, or those raising cats or dogs. By multivariate analysis, FBT infection was only related to the consumption of raw fish and gender. Seventy stool samples with a sufficient amount of faecal matter were subjected to DNA extraction, 42.85% of them yielded DNA production, and all were of* Clonorchis sinensis*.* Conclusion*. Results of the study showed the high prevalence of infection of fish-borne trematode, mostly* C. sinensis* among humans in Ninh Binh province. The prevention of FBT should be strengthened with programs detailed according to the distribution of FBT in different endemic areas.

## 1. Introduction

Digenetic trematodes (digeneans or flukes) are commonly called “flatworms” and a major group of human parasites. They are classified as liver, lung, intestinal, or blood flukes according to the typical microhabitat in which the adult parasite usually resides. Some trematodes such as small liver flukes (SLF) (*Clonorchis sinensis/Opisthorchis viverrini*) and minute intestinal flukes (MIF) (*Haplorchis pumilio; H. taichui*) are of medical importance and public health significance in Asia [1]. Life cycles of FBT involve three types of hosts that are firstly aquatic snail hosts, secondary fish hosts, and definitive hosts including a range of fish-eating mammals and birds [2]. For the same mode of transmission, SLF and MIF infection may coexist in regions where local people have a habit of eating raw fish including Lao PDR, northern Thailand, and Vietnam [3–8]. Although they share many similar biological aspects, SLF and MIF differ in terms of intermediate hosts, time to complete life cycle in the definite host and life expectancy in the human body, response to antihelminth drugs, etc. [9–11]. Thus, control measures have to be appropriately adjusted according to the distribution of different species in endemic areas.

Vietnam is a South East Asian country with the existence of many parasitic zoonoses [12]. Results of epidemiological surveys showed that SLF* (Clonorchis sinensis)* and some MIF (*Haplorchis pumilio, H. taichui, H. yokogawai, *and* Stellantchasmus falcatus*) were endemic in many northern provinces including Ninh Binh province, but there is still controversy over the distribution of these flukes among local people [4, 13–18]. The diagnosis of FBT infection in human has been mostly based on morphological features of small fluke eggs in stool samples [19], but due to the similarity of these eggs, the precise discrimination is nearly impossible [20, 21]. The molecular technique is a rapid and highly sensitive tool to identify FBT eggs but has not been applied in the previous surveys. Studies using molecular tools to identify the species of FBT for adult worms collected from infected persons have revealed inconsistent results [15, 18]. For a long time, much attention has focused on SLF [19, 22] but most of the metacercariae collected from fish in that region were MIF while the prevalence of SLF is very low [17, 23, 24]. With such divergent results, FBT infection among local people should be resituated. The present study was carried out to investigate the prevalence and distribution of FBT species in human as well as factors that affected the transmission of FBT in this endemic area of Vietnam.

## 2. Material and Methods

### 2.1. The Site, Sampling, and Examination Procedure

**Figure 1:**
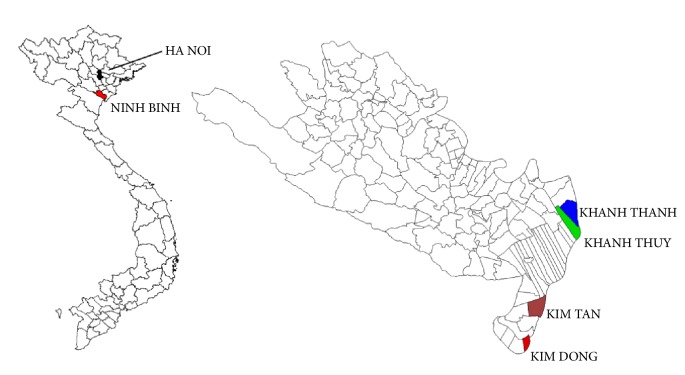
The study site.

The cross-sectional survey was conducted in four communes, Kim Dong and Kim Tan of Kim Son district and Khanh Thanh and Khanh Thuy of Yen Khanh district, Ninh Binh province (Figure 1). The study site is located around 100 km southeast of the capital, Hanoi. Most residents of the four communes live on rice agriculture, while some residents of Kim Tan commune (Kim Son district)—a coastal commune—work as fish farmers.

The sample size for this study is determined by the standard formula (n=*z*_1−*α*/2_^2^p(1-p)/d^2^) to reach a universal sampling size. At 95 percent confidence interval, absolute precision (d) of 5% and anticipated population proportion (p) of 50%, the desired sample size was 384 [25].

Households in these four communes were randomly selected from the lists provided by local health authorities. In each selected household, all members aged 15 years or older were chosen for the study. About one hundred persons from each commune were involved and a total of 400 participants completed a questionnaire about their demographic features and some habits such as consuming raw fish or drinking alcohol. The respondents were provided with labelled bottles to collect stool samples and required to transport the samples on the same or the following day. The stool samples were placed in dry ice boxes and transported to the laboratory to be examined. Helminth parasite examination was performed on the same day or the next day of receiving the stool samples. One gram of each stool sample was weighed and then tested for helminth eggs using formaldehyde–ether sedimentation technique [26]. All trematode eggs with sizes of less than 50 *μ*m were considered “small trematode eggs”. All the discovered eggs were recorded and the intensity of small trematode infection was categorized as light (< 1.000 eggs per gram (EPG)), moderate (1.000-10.000 EPG), or heavy infection (>10.000 EPG) [27]. One part of each positive sample was diluted in 3 parts by volume of alcohol (70%) and stored in -20°C for further analysis.

### 2.2. Molecular Analysis

The extraction of DNA from trematode eggs

Seventy positive samples with a sufficient amount of faecal matter were subjected to DNA extraction. For each stool sample, 1.4 ml of ASL buffer was added to 200 *μ*l faecal liquid, mixed continuously for 1 min or until the stool samples were thoroughly homogenized. After homogenization, the suspension was heated at 95°C for 4 min and then frozen in dry ice for 8 min. The freeze-thaw cycle was repeated twice before incubating at 95°C for 10 min. Then 1.2 ml of supernatant was collected into a new sterile tube. The DNA was extracted from the supernatant using the QIAmp DNA stool mini kit (QIAGEN, Hilden, Germany) according to the manufacturer's protocol. At the final step, DNA was eluted with 50 *μ*l of elution buffer.

Primers to amplify the second internal transcribed spacer region (ITS-2) included ITS2-F (5′-CTT GAACGC ACA TTG CGG CCA TGG G-3′) and ITS2-R (5′-GCG GGT AAT CAC GTC TGA GCC GAG G-3′) [28]. PCR reaction was conducted on a thermal cycler (Eppendorf Mastercycler Personal, Germany) in a total volume of 20 *μ*l, including 5 *μ*l template, 10 pmol of each primer and PCR master mix (PCR Master Mix from QIAGEN). The PCR was run 35 cycles of 94°C for 10 seconds, 40°C for 30 seconds, and 72°C for 30 seconds with a final extension step of 15 minutes at 72°C. PCR products were separated by electrophoresis on 1.2% agarose gel and visualized under UV light after staining with ethidium bromide. All yielded PCR products were purified and sequenced using a 3130XL sequencer. The obtained sequences of ITS2 region were aligned with reference sequences retrieved from GenBank using Bioedit 7.0 and MEGA 7 software [29]. Phylogenetic trees were constructed using the neighbor-joining method and significant level was estimated with 1000 bootstrap replicates.

Data were analyzed with the Statistical Package for Social Science (SPSS version 16.0). The comparison between numeric variables was done by student T-test. Univariate analysis of the relationship between prevalence and risk factors (living condition, gender, the habit of eating raw fish or alcohol) was conducted. Factors that showed a significant association with the small trematode egg-positive rate were then subjected to multiple logistic regression analysis. The p-value smaller than 0.05 was considered significant.

Time of the study: interviewing, sampling, and microscopic examination of stool samples were done between March 2016 and March 2017. Molecular analysis was conducted in 2018.

*Ethical Consideration*. The study was approved by the ethical committee of the National Institute of Malaria, Parasitology and Entomology of Vietnam. Written consent was obtained from all subjects and all persons with positive results of parasite infection were provided free drug treatment at local health care service.

## 3. Results

Four hundred people with an average age of 46.8 years old were involved in the present study, 61% of them were male and most of them were farmers with a low level of education, none of them having graduated from a university.

Among the participants, 19.5% were infected with FBT. By univariate analysis, persons who were men, eating raw fish and drinking alcohol were at a higher risk of infection (OR = 3.994, 6.769 and 2.680, respectively). By multivariate analysis, only gender and habit of eating raw fish were the risk factors of getting FBT infection (Table 1).

There was no relationship between house characteristics, raising dogs or cats, and FBT infection (Table 2).

The average density of infection was 517.06 EPG. Most of the infected participants were ranked as light infection (87.17%) and mean of EPG were higher among male and those who ate raw fish and drank alcohol (Table 3).

**Figure 2:**
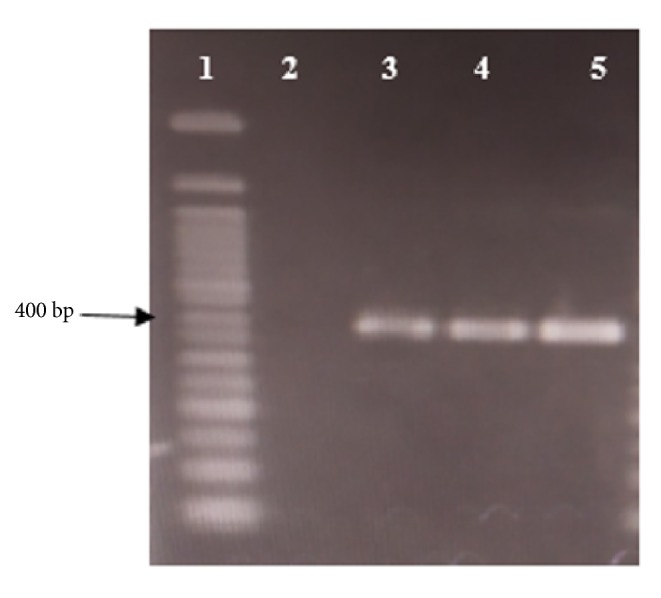
The products of the amplification of the small trematode eggs. Lane 1: 50 bp DNA marker (ThermoFisher), Lane 2: negative control, and Lanes 3-5: positive samples.

Identification of trematode: thirty of 70 analyzed stool samples (42.85%) were ITS2-PCR positive with the sizes of 400 bp (Figure 2).

**Figure 3:**
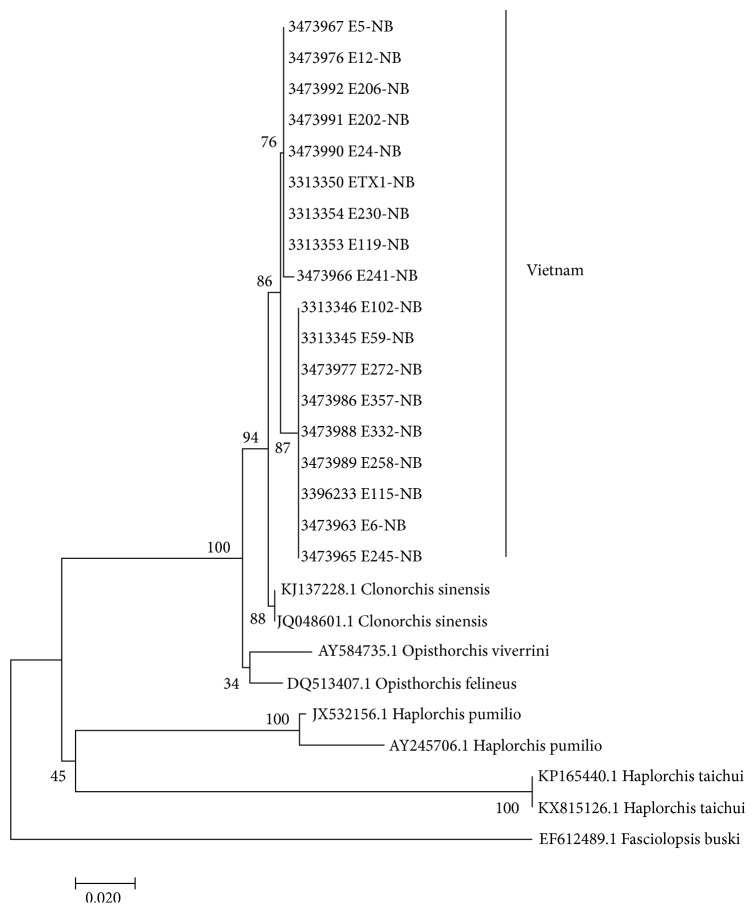
Neighbor-joining phylogenetic tree to identify trematode species.

The NJ phylogenetic tree was constructed from typing sentences of 18 representatives from our study (those with subfix NB in the tree) and 9 reference sequences from GenBank using CLUSTAL_X with Kimura's correction. All the obtained sequences in our study were determined as* Clonorchis sinensis *and none were MIF (Figure 3). Some sequences were deposited in GenBank under accession number MK453253, MN128615, MN128616, MN128617, and MN128618.

## 4. Discussion

In Vietnam, FBT infection is endemic in the north and the highest prevalences were recorded in Nam Dinh and Ninh Binh provinces [19]. The prevalence of FBT in Kim Son and Yen Khanh districts, Ninh Binh province (19.5%), was lower than that of a previous survey carried out in Kim Son district between 1999 and 2000 [15] (26.1%) but higher than a report in Gia Vien, a nearby district in 2015 (16.47%) [30]. The average intensity of infection was 517 eggs per gram which was comparable to that in previous reports in Kim Son district (mean of 504 and 472 EPG) [13, 15]. Almost all (87.2%) cases were light infection and no subjects were heavily infected. This finding was consistent with some observations suggesting that most of the persons infected with small fluke in the community were categorized as light infection [4, 31].

Eating raw fish was the leading factors of FBT infection among local people (OR = 6.769), which was consistent with some other reports [30, 32–37]. Nearly three-quarters of participants usually consumed raw fish which was comparable to the result of Thach et al. (2008) [15] and Vinh et al. (2017) [30]. Some authors stressed that the control of FBT is theoretically very simple by avoiding eating raw or undercooked fish, but it can be extremely difficult in facing of centuries-old traditions [38, 39]. Although many efforts had been made to change the habit of local people, the rate of consuming raw fish was almost unchanged and more works are required to deal with this situation. The prevalence and intensity of FBT infection among males being higher than in women were in line with results of some other studies [14, 32, 40]. By univariate analysis, people who are alcohol drinkers were at 2.680 times higher risk of getting infected than those who did not drink alcohol but by multivariate analysis, drinking alcohol was not related to FBT infection. This may be because it is a common habit of local people to drink alcohol while eating raw fish. This means the main factor for infection is the consumption of raw fish, and drinking is only a confounding factor. However, alcohol drinkers had higher EPG mean compared to that of nondrinker (Table 3) and there is an experiment showing that alcohol could induce metacercariae excystation, leading to the early development of trematodes in human [41].

There was no risk of increased infection among local people living at home without hygienic latrines, those living close to rivers or having ponds, or those raising cats or dogs. The relationship between these factors and FBT infection has been reported in some studies. Higher prevalence of FBT infection among people living in lowland areas [14, 42]; near freshwater sources [43]; or fish ponds [36] was noted. Nevertheless, there is only a weak relationship between these factors and FBT infection in the above reports and Tesana S et al. (1991) noted a higher prevalence of SLF infection among the people residing far from the rivers than those residing on the banks [44]. Dogs and cats are considered the reservoirs of FBT flukes [45] and the high prevalence of* C. sinensis* in cats and dogs corresponded to high prevalence in humans in South China [46]; therefore controlling flukes in animals may play a role in preventing human infection [12]. However, FBT could not transmit to human by direct contacts with animals, so the absence of a relationship between having dogs or cats and FBT infection was reasonable.

In the present study, molecular techniques were performed to determine the distribution of FBT species among local people and this may be the first report using the molecular technique to discriminate eggs of FBT in Vietnam. Internal transcribed spacer region was selected because it is considered a reliable and precise marker for identification of flukes [44]. Less than half of the analyzed stool samples yielded DNA production. The low sensitivity of molecular techniques is probably related to the low density of eggs in faeces which has been documented by some authors [47, 48]. All yielded DNA products belonged to* C. sinensis* which is consistent with some other reports in northern Vietnam. Dang el al. (2008) found that all adult worms collected from infected people are* C. sinensis *[15]. De and Hoa (2011) showed that among 10 infected persons there were 9 persons infected with* C. sinensis* and 10 persons infected with MIF [18]. Notably, results of the present study contradicted a survey carried out by Dung et al. (2007) which found a high rate of MIF (100%) and lower prevalence of SLF (about 50%) among infected persons [4]. However, previous studies were based on analysis of adult flukes collected from a heavily infected person after taking drugs which meant for intentionally selected persons. Our study is based on molecular analysis of all infected people so that the result would be more representative of the community.

The predominance of* C. sinensis* among people in the present study did not agree with the predominance of MIF (such as* Haplorchis pumilio*) among fish in northern Vietnam [17, 23, 24]. There may be some possible explanation for this contradiction. The first may be the difference between the longevity of two kinds of fluke in humans. In humans,* C. sinensis* can live for 26 years [49] but most MIF only live for one year [50] so the accumulation rate of infection with SLF for such a long time is very high. The second reason is the productivity of different flukes. One SLF can produce up to 4000 eggs per day [51] while the daily egg output of some MIF such as* H. taichui *is very low (estimated as 82 eggs/worm) [52]. This may have led to the difference in intensity of infection, which affected the sensitivity of diagnosis technique based on molecular analysis.

Our findings would be useful in adjusting the response to FBT infection in that area especially measures to reduce the worm burden or morbidity rate by chemotherapy. Praziquantel is the most common means for large-scale or individual treatment [53] and this approach must be based on the results of epidemiological studies [27]. With almost all positive persons infected with* C. sinensis* and the low efficacy of praziquantel on clonorchiasis in the north of Vietnam [9], closely monitoring the efficacy of praziquantel at the community level is needed and a modified dose accordingly may be considered in some selected population.

## 5. Conclusion

The study aims to explore the prevalence of different species of FBT and related factors among local people in a northern province of Vietnam. The prevalence of infection of fish-borne trematode among human in Kim Son and Yen Khanh district, Ninh Binh province, was still high although most were of a light infection and infected with* Clonorchis sinensis*. The main risk factor of infection was the common habit of eating raw fish by local people. There is a need to strengthen the prevention of FBT in endemic areas including better targeted public education interventions about FBT and people at risk. Applying modern techniques to accurately identify the fluke in community surveys in different areas to get precise information of epidemiology fish-borne disease is very important to adjust response measures to control the infection.

## Figures and Tables

**Table 1:** Prevalence and related factors of small trematode infection.

		N	Infected	%	Univariate analysis	Multivariate analysis
					OR (CI 95%)	OR (CI 95%)
Districts	Kim Son	199	40	20.10	1.079 (0.658 – 1.770)	
	Yen Khanh	201	38	18.91		
Gender	Male	244	65	26.64	3.994 (2.117 – 7.536)	5.088 (1.766 – 14.660)
	Female	156	13	8.33		
Age groups	15 –<30	30	5	16.67		
	30 – <40	70	13	18.57		
	40 – < 50	120	22	18.33		
	50 – < 60	132	30	22.73		
	≥ 60	48	8	16.67		
Eating raw fish	Yes	293	73	24.91	6.769 (2.655 – 17.259)	5.529 (2.066 – 14.798)
	No	107	5	4.67		
Drink alcohol	Yes	267	64	23.97	2.680 (1.440 – 4.986)	0.448 (0.151 – 1.325)
	No	133	14	10.53		
Total		400	78	19.50		

**Table 2:** Some other factors related to small trematode infection.

		Infected	Not-infected	OR (CI 95%)
House with hygienic latrines *∗*	No	15	36	1.892
	Yes	63	286	(0.976 – 3.664)
House nearby rivers*∗∗*	Yes	57	212	1.408
	No	32	140	(0.812 – 2.443)
House with fish ponds	Yes	46	182	1.106
	No	32	140	(0.669 – 1.827)
Raising dogs	Yes	57	229	1.102
	No	21	93	(0.633 – 1.921)
Raising cats	Yes	48	205	0.913
	No	30	117	(0.549 – 1.520)

*∗*Hygienic latrines: septic tank.

*∗∗*House nearby rivers: distance less than 1 km from rivers.

**Table 3:** Intensify of small trematode infection and related factors.

Groups		N (%)	Mean (EPG)	SD	p
Whole			517.06	1103.49	
	Light	68 (87.17)			
	Moderate	10 (12.83)			
	Heavy	0 (0)			
District	Kim Son	40	723.00	1464.42	> 0.05
	Yen Khanh	38	396.84	466.51	
Age groups	15 –<30	5	560.00	689.35	> 0.05
	30 – <40	13	264.62	164.55	
	40 – < 50	22	458.18	545.49	
	50 – < 60	30	853.33	1665.10	
	≥ 60	8	260.00	177.60	
Gender	Male	65	618.46	1199.98	< 0.05
	Female	13	292.31	194.17	
Eating raw fish	Yes	73	587.39	1136.54	< 0.05
	No	5	224.00	186.76	
Drinking alcohol	Yes	47	788.94	1375.68	< 0.05
	No	31	223.23	155.87	

## Data Availability

The datasets used and/or analyzed during the current study are available from the corresponding author upon reasonable request.

## Abbreviations

CI: Confidence interval
DNA: Deoxyribonucleic acid
dNTP: Deoxynucleotide
EPG: Eggs per gram
FBT: Fish-borne trematodes
ITS: Internal transcribed spacer
MIF: Minute intestinal flukes
OR: Odds ratios
PCR: Polymerase chain reaction
SLF: Small liver flukes.
